# The Small RNA ErsA Impacts the Anaerobic Metabolism of *Pseudomonas aeruginosa* Through Post-Transcriptional Modulation of the Master Regulator Anr

**DOI:** 10.3389/fmicb.2021.691608

**Published:** 2021-08-20

**Authors:** Silvia Ferrara, Riccardo Carrubba, Silvia Santoro, Giovanni Bertoni

**Affiliations:** Department of Biosciences, Università degli Studi di Milano, Milan, Italy

**Keywords:** *Pseudomonas aeruginosa*, small RNA, Anr, cystic fibrosis, anoxia adaptation

## Abstract

*Pseudomonas aeruginosa* is one of the most critical opportunistic pathogens in humans, able to cause both lethal acute and chronic lung infections. In previous work, we indicated that the small RNA ErsA plays a role in the regulatory network of *P. aeruginosa* pathogenicity in airways infection. To give further insight into the lifestyle functions that could be either directly or indirectly regulated by ErsA during infection, we reanalyzed the categories of genes whose transcription appeared dysregulated in an *ersA* knock-out mutant of the *P. aeruginosa* PAO1 reference strain. This preliminary analysis indicated ErsA as a candidate co-modulator of denitrification and in general, the anaerobiosis response, a characteristic physiologic state of *P. aeruginosa* during chronic infection of the lung of cystic fibrosis (*CF*) patients. To explain the pattern of dysregulation of the anaerobic-lifestyle genes in the lack of ErsA, we postulated that ErsA regulation could target the expression of Anr, a well-known transcription factor that modulates a broad regulon of anoxia-responsive genes, and also Dnr, required for the transcription activation of the denitrification machinery. Our results show that ErsA positively regulates Anr expression at the post-transcriptional level while no direct ErsA-mediated regulatory effect on Dnr was observed. However, Dnr is transcriptionally downregulated in the absence of ErsA and this is consistent with the well-characterized regulatory link between Anr and Dnr. Anr regulatory function is critical for *P. aeruginosa* anaerobic growth, both through denitrification and fermentation of arginine. Interestingly, we found that, differently from the laboratory strain PAO1, ErsA deletion strongly impairs the anaerobic growth by both denitrification and arginine fermentation of the RP73 clinical isolate, a multi-drug resistant *P. aeruginosa CF*-adapted strain. This suggests that *P. aeruginosa* adaptation to *CF* lung might result in a higher dependence on ErsA for the transduction of the multiple signals to the regulatory network of key functions for survivance in such a complex environment. Together, our results suggest that ErsA takes an upper place in the regulatory network of airways infection, transducing host inputs to biofilm-related factors, as underlined in our previous reports, and to functions that allow *P. aeruginosa* to thrive in low-oxygen conditions.

## Introduction

The small RNA (sRNA) ErsA of *Pseudomonas aeruginosa* is associated with the regulation of bacterium-host interaction traits, such as biofilm maturation, motility (Falcone et al., 2018), and resistance to carbapenem antibiotics (Zhang et al., 2017; Sonnleitner et al., 2020). A key role of ErsA in the regulatory network of *P. aeruginosa* pathogenicity was recently assessed (Ferrara et al., 2020). The deletion of ErsA leads to *P. aeruginosa* virulence attenuation both *in vitro* and *in vivo* (Ferrara et al., 2020). The significant impairment of *P. aeruginosa* in biofilm formation and maturation resulting from ErsA deletion can explain the involvement of ErsA in acute infection and immune response activation (Ferrara et al., 2020). The ErsA role in biofilm regulation is supposed to involve the downregulation of the AlgC enzyme (Ferrara et al., 2015) and the activation of the AmrZ regulon (Falcone et al., 2018). However, the ErsA role in the host-pathogen interaction is not only limited to biofilm regulation but influences also the envelope composition of *P. aeruginosa* by direct negative regulation of *oprD* mRNA (Zhang et al., 2017; Sonnleitner et al., 2020). Moreover, recent transcriptomics data (Falcone et al., 2018) suggest a broader regulatory network influenced by ErsA since the ErsA deletion can affect other aspects of *P. aeruginosa* lifestyle when coping with, for example, an oxygen-restricted environment, such as the airways of cystic fibrosis (*CF*) patients (Govan and Deretic, 1996; Worlitzsch et al., 2002).

The complex matrix of biofilm structure and thick mucus in *CF* lungs impede oxygen diffusion and generate a hypoxic microenvironment. It was shown that microaerophilic and anaerobic conditions are predominant in the sputum of *CF* patients (Yoon et al., 2002; Alvarez-Ortega and Harwood, 2007; Hassett et al., 2009). It has been known for a long time that *P. aeruginosa, being a facultative anaerobe, can thrive in such CF* environment within mucus plugs and biofilm (Schobert and Jahn, 2010) and besides anoxia and hypoxia are thought to be essential for full biofilm establishment (Stewart and Franklin, 2008).

In case of limiting or no oxygen availability, denitrification allows *P. aeruginosa* to respire nitrates or nitrites (Carlson and Ingraham, 1983; Davies et al., 1989; Zumft, 1997). When these are also absent, *P. aeruginosa* can moderately support anaerobic growth and survival through the activation of the arginine fermentation pathway (Vander Wauven et al., 1984; Luthi et al., 1990) and pyruvate fermentation (Eschbach et al., 2004; Supplementary Figure S1, schematizing the complex anaerobic metabolism of *P. aeruginosa*).

The expression of the enzymes for growth and survival in anaerobic environments is coordinated by the transcriptional factor Anr, a global oxygen-sensing transcription factor that plays the role of the master regulator of anaerobiosis-related genes (Galimand et al., 1991; Zimmermann et al., 1991; Ye et al., 1995; Platt et al., 2008; Trunk et al., 2010). Anr is essential for *P. aeruginosa* anaerobic growth on both nitrate and arginine (Filiatrault et al., 2006) and responsible for the activation of the *ackA-pta* operon for pyruvate fermentation in response to oxygen limitation (Eschbach et al., 2004; Filiatrault et al., 2006). Moreover, it controls the expression of *dnr* and *narL* genes, encoding two transcription factors required for the activation of the denitrification machinery and the regulation of other genes linked to anaerobic metabolism (Schreiber et al., 2007; Arai, 2011; Tribelli et al., 2019).

In this work, we expand the knowledge on the regulatory network of ErsA and show its additional role in the regulation of Anr and consequently of anaerobic metabolism and denitrification processes. Therefore, ErsA is not only correlated to anaerobiosis as transcriptionally activated under reduced oxygen conditions (Ferrara et al., 2015), but it also transduces the low-oxygen cue toward the Anr regulon acting as a positive post-transcriptional regulator of the *anr* mRNA. Furthermore, we show that, beyond a certain threshold of ErsA abundance, the RNA-binding protein Hfq cooperates with ErsA in Anr activation. This role of Hfq is added to the one exercised post-transcriptionally *per se* by Hfq on Anr. Finally, ErsA deletion strongly impairs the anaerobic growth by both denitrification and arginine fermentation of the RP73 clinical isolate, a multi-drug resistant *P. aeruginosa CF*-adapted strain. This suggests that *P. aeruginosa* adaptation to *CF* lung might result in a higher dependence on ErsA for the regulation of anaerobic metabolism.

## Materials and Methods

### Bacterial Strains and Culture Conditions

Bacterial strains and plasmids used in this study are listed in Supplementary Table S1. *P. aeruginosa* and *E. coli* strains were routinely grown in Luria-Bertani broth (LB) at 37°C. For selective *E. coli* growth, ampicillin, gentamicin, and chloramphenicol were added at 100, 20, and 25μg/ml, respectively. For selective *Pseudomonas* growth, carbenicillin and gentamicin were added at 300 and 60μg/ml, respectively. For P_BAD_ induction in vector plasmid pGM931, arabinose was added to a final concentration of 10mm.

Anaerobic cultivations of *P. aeruginosa* strains were performed in Oxoid anaerobiosis jars at 37°C, using agar plates prepared with Brain Heart Infusion (BHI)-rich medium supplemented with 100mm KNO_3_ to allow anaerobic respiration or without KNO_3_ to test arginine fermentation. For the anaerobic growth assay, cell cultures of *P. aeruginosa* RP73 (Bianconi et al., 2015) and RP73 Δ*ersA* (Ferrara et al., 2020), PAO1 (Stover et al., 2000), and PAO1 Δ*ersA* (Ferrara et al., 2015) with an OD_600_ of 1 (corresponding to 1×10^9^CFU/ml) were serially diluted until 10^−6^; 2μl of each dilution was spotted and incubated for 72h. For total RNA extraction from anaerobically grown cell cultures of PAO1 and PAO1 Δ*ersA* (Ferrara et al., 2015), the strains were plated at the confluence and incubated for 72h. The anaerobic atmosphere was induced by the Oxoid AnaeroGen sachet. Control testing of anaerobiosis was performed using the Oxoid Anaerobic indicator in the jar as a visual check that anaerobic conditions have been achieved and maintained.

### Plasmid Constructions

The oligonucleotides used in this study are listed in Supplementary Table S2. Plasmids pBBR1-*anr::sfGFP* and pBBR1-*dnr::sfGFP* expressing *anr::sfGFP* and *dnr::sfGFP* translational fusions, respectively, under the PLtetO-1 constitutive promoter were constructed as follows. A DNA fragment including the 31-nt UTR along with the first 22 codons (66nt) of the *anr* open reading frame (66nt) was PCR amplified from PAO1 genomic DNA with oligos 2/3, digested with *Nsi*I/*Nhe*I, and cloned into the sfGFP reporter vector pXG10-SF (Corcoran et al., 2012) giving rise to plasmid pXG10-*anr::sfGFP*. With the same procedure, a DNA fragment including the 116-nt UTR and the first 32 codons (96nt) of the *dnr* open reading frame was amplified with oligos 4/5, digested *Nsi*I/*Nhe*I, and cloned into the sfGFP reporter vectors pXG10-SF giving rise to plasmid pXG10-*dnr::sfGFP*. The DNA fragments spanning from the PLtetO-1 promoter to the end of the *sfGFP* reporter gene were amplified by PCR, respectively, from pXG10-*anr::sfGFP* and pXG10-*dnr::sfGFP* with oligos 6/7, digested *Cla*I/*Xba*I, and cloned into the low-copy number shuttle vector pBBR1-MCS5 (Kovach et al., 1995) giving rise to constructs pBBR1-*anr::sfGFP* and pBBR1-*dnr::sfGFP*, respectively. All constructs were verified by sequencing (Eurofins Genomics) using either oligos 7 or 8.

### *In vitro* Assays of sRNA/mRNA Interactions

Purified RNA for RNA/RNA interaction assays was prepared by T7 RNA polymerase transcription of gel-purified DNA fragments. DNA fragments for *anr* mRNA and ErsA RNA preparations were amplified from *P. aeruginosa* PAO1 genomic DNA with oligo pairs 9/10 and 11/12, respectively. Each transcription reaction was performed with the Riboprobe^®^ System-T7 (Promega) with 300ng of DNA template. Synthesized RNA was purified using the RNeasy MinElute Cleanup Kit (Qiagen). Purified RNA was checked by denaturing polyacrylamide gel electrophoresis and quantified using Eppendorf Biospectrometer. Electrophoretic Mobility Shift Assay to analyze ErsA/*anr* mRNA interactions was performed in 10μl of reactions containing 1×RNA-binding buffer (10mm Tris–HCl, pH 7, 100mm KCl, 10mm MgCl_2_, and 10% glycerol), purified ErsA RNA and increasing amounts of purified *anr* mRNA, or yeast tRNA (Ambion). Binding reactions were incubated at 37°C for 20min, then loaded into a native 6% polyacrylamide gel (acrylamide-bis ratio 29:1) in 0.5×TBE buffer (45mm Tris-borate, pH 8.0, 1mm EDTA), and electrophoresed using a Mini-Protean Electrophoresis System (Bio-Rad) at 4°C and 180V for 90min. RNAs were transferred into a GeneScreen plus nylon hybridization transfer membrane (Perkin Elmer) using a semi-dry electroblotting (Fastblot B33, Biometra) set at 25V, 400mA for 1h, and UV-crosslinked to the membrane with a Stratalinker 1800 UV Crosslinker (Stratagene). The blotting membrane was hybridized with a biotinylated anti-ErsA probe (oligo 1) using the North2South Chemiluminescent Hybridization and Detection kit (Thermo Scientific) according to the manufacturer’s instructions. After the addition of the Streptavidin-HRP conjugate, the ErsA bands were visualized by mixing equal volumes of luminol/enhancer solution and stable peroxide solution and acquiring images with a ChemiDoc Touch Imaging System (Bio-Rad) using ImageLab analysis software.

### *In vivo* Assays of sRNA/mRNA Interactions

Fluorescence measurements in *P. aeruginosa* strains PAO1 wild type (Stover et al., 2000), PAO1 Δ*ersA* (Ferrara et al., 2015), and PAO1 *hfq*^−^ (Sonnleitner et al., 2003) carrying the sfGFP translational fusions with target genes were carried out as follows. To test the sRNA/target interaction in planktonic cells of aerobically grown cultures, strains carrying the reporter pBBR1-*anr::sfGFP* or pBBR1-*dnr::sfGFP* alone, or combined with either pGM931 (Qiu et al., 2008; Delvillani et al., 2014; Ferrara et al., 2020) or pGM-*ersA* (Ferrara et al., 2015), were inoculated in 15ml tubes filled with 5ml of LB at an OD_600_ of 0.1 and grown at 37°C in a rotatory shaker. Samples were taken after 6 and 24h (Ferrara et al., 2015). For the testing of anaerobically grown cultures, strains were inoculated at an OD_600_ of 0.4 in 50ml flasks filled with 10ml of LB medium supplemented with 100mm KNO_3_. The anaerobic atmosphere was induced by Oxoid AnaeroGen sachet. Control testing of anaerobiosis was assessed by the Oxoid Anaerobic Indicator. Samples were taken after 2days of static anaerobic growth at 37°C. The collected samples were centrifuged, washed twice, and resuspended in PBS (10mm Na_3_PO_4_, 150mm NaCl) to OD_600_ of 1. Samples were then serially diluted 1.33-fold (corresponding to OD_600_ of 0,75, 0.5, and 0.25). 200μl of aliquots was transferred to black polystyrene 96-well microplates with a clear, flat bottom (Corning). At least three biological replicates were used for every experimental set. The absorbance (Abs_595_) and fluorescence polarization (FP_485/535_) were measured in an EnSight Multimode Plate Reader (PerkinElmer) using Kaleido data acquiring software. GFP activity was expressed in arbitrary units (AU) as FP_485/535_/Abs_595_.

To test fluorescence in surface-grown cells, the strains were spotted on agar plates of BHI supplemented with or without 100mm KNO_3_ and incubated at 37°C for 72h. Cells were collected from spots, resuspended in PBS, and assayed as described above.

### RNA Isolation and Quantitative RT-PCR Analysis

Quantitative RT-PCR analysis (qRT-PCR) was performed on total RNA extracted from *P. aeruginosa* PAO1 wild type and Δ*ersA* surface-grown cells under anaerobic conditions for 72h. Immediately after opening the jar, cells were removed from plates, resuspended in RNAprotect Cell Reagent (Qiagen), incubated, for 5min at room temperature, pelleted by centrifugation, and stored at −80°C until use. RNA extraction was performed as described previously in Ferrara et al. (2015). The quality and concentration of the extracted RNA were assessed by a Biospectrometer (Eppendorf).

cDNA was synthesized from 1μg of total purified RNA using Superscript III Reverse Transcriptase (Invitrogen) according to the manufacturer’s instructions. qRT-PCR was performed in triplicate using SYBR Green PCR Master Mix (Bio-Rad) on a CFX Connect Real-Time System (Bio-Rad). Oligo pairs 13/14 (Vitale et al., 2008), 15/16 (Tribelli et al., 2019), and 17/18 (Jackson et al., 2013) were used for amplification of 16S, *anr*, and *dnr*, respectively. The reaction procedure involved incubation at 95°C for 5min and 39cycles of amplification at 95°C for 15s, 58°C for 20s, and 72,5°C for 30s. The calculation of the relative expression of *anr* and *dnr* genes in the PAO1 Δ*ersA* mutant vs. the wild-type strain was performed first normalizing mRNA amounts to 16S ribosome RNA (Δ*C*_T_) and then relating the Δ*C*_T_ in the Δ*ersA* mutant to the wild type (ΔΔ*C*_T_).

## Results

### The Regulation of Denitrification and Anaerobiosis in *P. aeruginosa* Is a Target of ErsA RNA

The transcriptome profiling of the aerobically grown PAO1 wild-type strain vs. the corresponding Δ*ersA* mutant showed more than 160 differentially expressed genes (DEGs) (Falcone et al., 2018). These data were collected from bacterial cells incubated at 37°C until the onset of the stationary phase (*OD*_600_ =2.7; Falcone et al., 2018).

For a more comprehensive understanding of the ErsA-dependent regulatory networks, we performed a new functional categorization of the above-mentioned DEGs. This analysis revealed 52 DEGs associated with growth or survival under anaerobic conditions (Table 1). Among these, some genes belong to the denitrification and nitrate metabolism (*narK1*, *narH*, *narI*, *narJ*, *narL*, and *nirN*), the fermentation pathways of arginine and pyruvate (*arcD*, *ackA*), and the universal stress response (*uspL*/PA1789, *uspM*/PA4328, *uspN*/PA4352, and *uspO*/PA5027). Out of 52 anaerobiosis-linked DEGs, 44 and 5 were already known to be up- or downregulated under anaerobic growth, respectively (Alvarez-Ortega and Harwood, 2007; Platt et al., 2008; Trunk et al., 2010; Crespo et al., 2017; Tribelli et al., 2019).

**Table 1:** Differentially expressed genes (DEGs) in the PAO1 wild-type vs. the Δ*ersA* mutant strains belonging to regulons responsive to anoxic conditions.

Locus	Description	Δ*ersA* effect^a^	Log_2_ (FC)^b^	Anaerobic response by^c^		References
				Anr	Low O_2_	
**Transcription regulators**						
PA1196	probable transcription regulator	D	−2.53	U	U	Alvarez-Ortega and Harwood, 2007; Tribelli et al., 2019
PA2127	*cgrA*, cupA gene regulator A CgrA	D	−1.57		U	Alvarez-Ortega and Harwood, 2007; Tribelli et al., 2019
PA2663	*ppyR*, psl and pyoverdine operon regulator PpyR	D	−2.18	U	U	Trunk et al., 2010
PA3006	*psrA*, transcription regulator PsrA	D	−1.35	U	U	Trunk et al., 2010
PA3458	probable transcription regulator	D	−1.97		U	Trunk et al., 2010
PA3879	*narL*, two-component response regulator NarL	D	−1.67		U	Trunk et al., 2010
PA3973	probable transcriptional regulator	D	−1.64		U	Trunk et al., 2010
PA4596	*esrC*, EsrC	D	−2.53		U/D	Alvarez-Ortega and Harwood, 2007; Tribelli et al., 2019
**Energy metabolism**						
PA3613	D-xylulose 5-phosphate phosphoketolase	D	−1.77		U	Alvarez-Ortega and Harwood, 2007; Tribelli et al., 2019
PA0509	*nirN*, NirN	U	2.22		U (and U in response to nitrate)	Alvarez-Ortega and Harwood, 2007; Platt et al., 2008
PA3872	*narI*, respiratory nitrate reductase gamma chain	U	3.08		U (and U in response to nitrate)	Alvarez-Ortega and Harwood, 2007; Platt et al., 2008
PA3873	*narJ*, respiratory nitrate reductase delta chain	U	2.19		U (and U in response to nitrate)	Alvarez-Ortega and Harwood, 2007; Platt et al., 2008
PA3874	*narH*, respiratory nitrate reductase beta chain	U	1.65		U (and U in response to nitrate)	Alvarez-Ortega and Harwood, 2007; Platt et al., 2008
**Transport**						
PA3465	major facilitator superfamily transporter	D	−1.57	U	U	Alvarez-Ortega and Harwood, 2007; Tribelli et al., 2019
PA3877	*narK1*, nitrite extrusion protein 1	D	−3.06		U	Alvarez-Ortega and Harwood, 2007; Platt et al., 2008; Tribelli et al., 2019
PA4610	copper transporter	D	−1.86	U	U	Trunk et al., 2010; Tribelli et al., 2019
PA5170	*arcD*, arginine/ornithine antiporter	D	−2.09	U	U	(Trunk et al., 2010; Tribelli et al., 2019)
PA5232	secretion protein HlyD family; glycoside hydrolase family 43	D	−1.29	U	U	Trunk et al., 2010; Tribelli et al., 2019
**Cell wall/LPS/capsule**						
PA3337	*rfaD*, ADP-L-glycero-D-mannoheptose 6-epimerase	D	−2.02	U	U	Trunk et al., 2010; Tribelli et al., 2019
**Membrane proteins**						
PA0563	membrane protein	U	1.50	D	D	Trunk et al., 2010
PA1429	probable cation-transporting P-type ATPase	D	−2.27		U	Alvarez-Ortega and Harwood, 2007; Tribelli et al., 2019
PA1337	*ansB*, glutaminase-asparaginase	D	−1.28		U	Alvarez-Ortega and Harwood, 2007; Tribelli et al., 2019
PA1546	*hemN*, oxygen-independent coproporphyrinogen III oxidase	D	−1.58	U	U	Trunk et al., 2010; Tribelli et al., 2019
PA1920	*nrdD*, class III (anaerobic) ribonucleoside-triphosphate reductase subunit, NrdD	D	−2.06		U	Alvarez-Ortega and Harwood, 2007; Crespo et al., 2017; Tribelli et al., 2019
**Antibiotic resistance**						
PA3614	metallo-β-lactamase superfamily protein	D	−1.56		U	Trunk et al., 2010; Tribelli et al., 2019
**Binding proteins**						
PA1673	Bacteriohemerythrin	D	−1.29	U	U	Trunk et al., 2010; Tribelli et al., 2019
PA4577	transfer protein TraR	D	−1.59	U	U	Trunk et al., 2010; Tribelli et al., 2019
**Degradation of chloroaromatic compounds**						
PA1597	dienelactone hydrolase	D	−1.52		U	Alvarez-Ortega and Harwood, 2007; Tribelli et al., 2019
**Chaperones and heat-shock proteins**						
PA3126	*ibpA*, heat-shock protein IbpA	D	−2.64		U	Trunk et al., 2010
PA4385	*groEL*, GroEL protein	D	−1.37		D	Alvarez-Ortega and Harwood, 2007; Tribelli et al., 2019
PA4760	*dnaJ*, DnaJ protein	D	−1.23		D	Alvarez-Ortega and Harwood, 2007; Tribelli et al., 2019
PA4761	*dnaK*, DnaK protein	D	−1.72		D	Alvarez-Ortega and Harwood, 2007; Tribelli et al., 2019
**Translation, post-translational modification, degradation**						
PA0579	*rpsU*, 30S ribosomal protein S21	U	1.41	D	D	Trunk et al., 2010
PA2619	*infA*, initiation factor	U	1.43	D	D	Trunk et al., 2010
PA4542	*clpB*, ClpB protein	D	−2.16		U/D	Alvarez-Ortega and Harwood, 2007; Tribelli et al., 2019
**Putative enzymes**						
PA0506	probable acyl-CoA dehydrogenase	D	−1.95	U	U	Trunk et al., 2010
PA0836	*ackA*, acetate kinase	D	−1.57		U	Trunk et al., 2010; Tribelli et al., 2019
PA2119	alcohol dehydrogenase (Zn-dependent)	D	−1.70	U	U	Trunk et al., 2010; Tribelli et al., 2019
PA2662	short-chain dehydrogenase	D	−2.03	U	U	Trunk et al., 2010
PA5475	Acetyltransferase	D	−1.65	U	U	Trunk et al., 2010; Tribelli et al., 2019
**Hypothetical proteins**						
PA0200	hypothetical protein	D	−1.26	U	U	Trunk et al., 2010; Tribelli et al., 2019
PA0526	DnrP	D	−1.96		U	Alvarez-Ortega and Harwood, 2007; Tribelli et al., 2019
PA1789	hypothetical protein	D	−1.57	U	U	Trunk et al., 2010; Tribelli et al., 2019
PA2753	hypothetical protein	D	−1.84	U	U	Trunk et al., 2010
PA2754	conserved hypothetical protein	D	−1.90	U	U	Trunk et al., 2010
PA2937	hypothetical protein	D	−1.93	U	U	Trunk et al., 2010
PA3572	hypothetical protein	D	−1.93		U	Trunk et al., 2010
PA4328	hypothetical protein	D	−1.44	U	U	Trunk et al., 2010; Tribelli et al., 2019
PA4352	conserved hypothetical protein	D	−1.42	U	U	Trunk et al., 2010; Tribelli et al., 2019
PA4387	*fxsA*, cytoplasmic membrane protein	D	−1.39		U	Trunk et al., 2010
PA5027	hypothetical protein	D	−2.00	U	U	Trunk et al., 2010
PA5446	hypothetical protein	D	−2.17		U	Trunk et al., 2010

a *D, downregulation; U, upregulation*

b *Log_2_ of Fold Change (FC) calculated as ratio of expression in ΔersA vs. wt*

c *D, downregulated; U, upregulated*.

The majority of the DEGs known to be induced by anaerobic conditions was downregulated in the Δ*ersA* strain. Vice versa, DEGs repressed in anaerobiosis and appeared upregulated in the Δ*ersA* strain. Exceptions are the *nirN* gene for nitrite respiration, and the last genes of the *nar* operon (*narH*, *J*, and *I*) for nitrate respiration, which are upregulated by anaerobiosis, and appear also upregulated in the Δ*ersA* mutant strain. Besides, as evidenced in Tables 1, 23 and 3 DEGs induced and repressed by anoxia, respectively, are under the transcriptional control of the master regulator of anaerobiosis, Anr (Trunk et al., 2010). Finally, the heat-shock protein IbpA and the transcription regulator PsrA (involved in the positive regulation of RpoS and the TTSS) were included among DEGs not belonging to the Anr regulon.

Taken together, this more accurate analysis of the comparative transcriptional profiling between PAO1 wild-type and *ΔersA* mutant strains suggested that the overall anaerobic metabolism in *P. aeruginosa* and particularly the denitrification processes could be co-regulated by ErsA. Despite the list of anaerobically regulated DEGs in Table 1 is reasonably not exhaustive since bacterial cells were grown in aerobic conditions before RNA extraction (Falcone et al., 2018), this evidenced that ErsA influences a peculiar set of genes belonging to the regulon of the transcription factor Anr. Specifically, the pattern of upregulated and downregulated genes in the PAO1 Δ*ersA* strain seemed to be consistent with positive post-transcriptional regulation of *anr* mRNA by ErsA. This suggested that Anr expression could be directly targeted by ErsA. Furthermore, since Anr is the master regulator in anaerobiosis of a broad regulon of anoxia-responsive genes (Platt et al., 2008; Trunk et al., 2010) including also the transcription factor DNR (Supplementary Figure S1), which is indeed responsible for the transcription activation of the denitrification machinery (Trunk et al., 2010), we speculated that ErsA might additionally regulate DNR expression.

### ErsA Targets *Anr* mRNA and Positively Regulates Anr Expression

**Figure 1:**
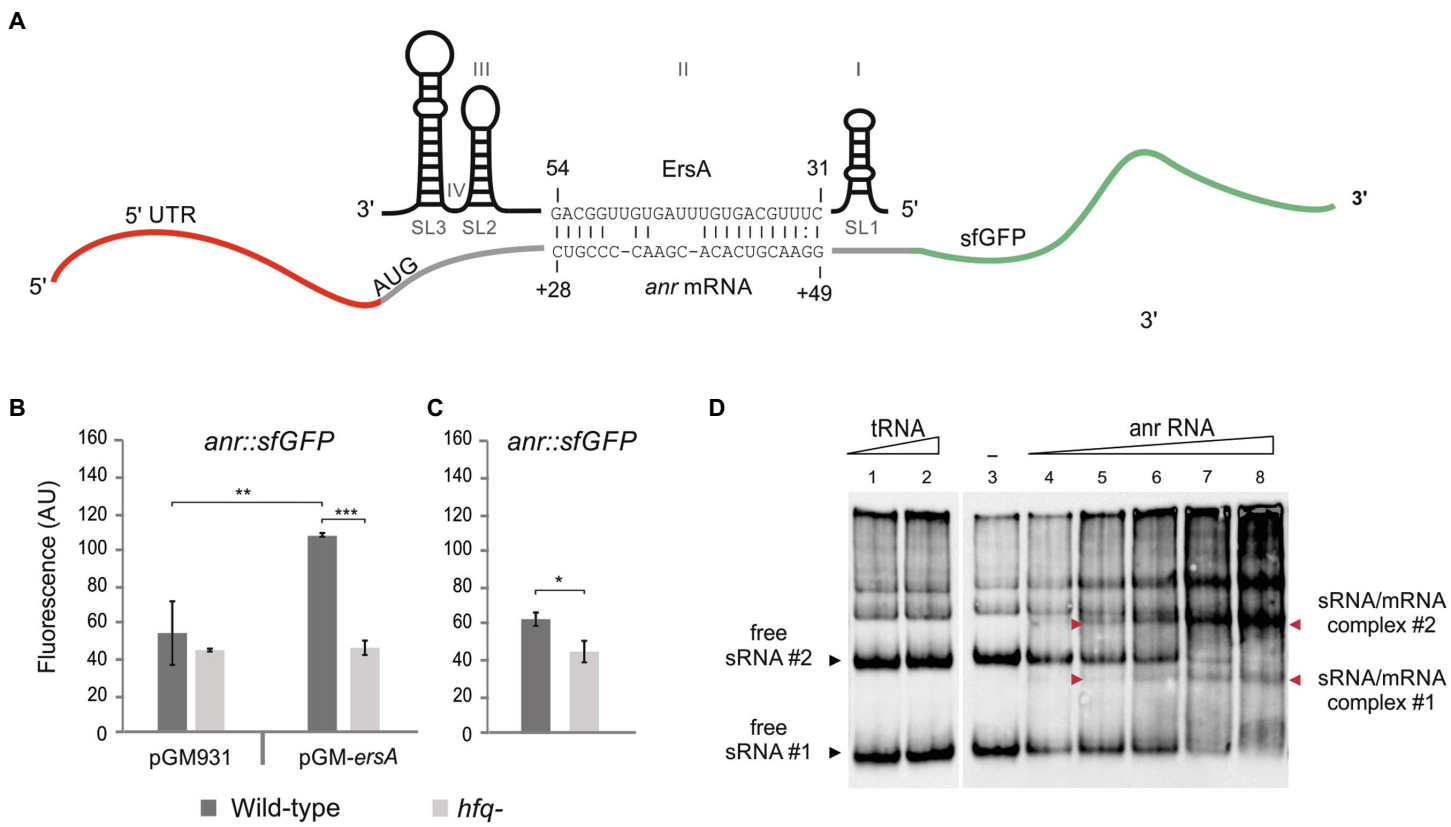
Interaction of ErsA with *anr* mRNA. **(A)** The sRNA ErsA consists of four domains named I, II, III, and IV, and three steam and loop structures called SL1, SL2, and SL3. ErsA is predicted to bind to *anr* mRNA using part of its U-rich unstructured domain II that locates between the two stem and loop structures SL1 and SL2. The ErsA region from nt 31 to nt 54 is predicted to bind within the coding sequence of *anr* mRNA, from nt +28 to +49 downstream the translation start site (ATG) by IntaRNA software. **(B)** Comparison of the fluorescence polarization expressed in arbitrary units (AU) resulting from the translational fusion *anr::sfGFP* in aerobically grown planktonic PAO1 wild-type (dark gray bars) and PAO1 *hfq^−^* (light gray bars) cells, in the absence (pGM931) and the presence (pGM-*ersA*) of ErsA overexpression. In the PAO1 wild-type background, ErsA overexpression from pGM-*ersA* results in an increase of the reporter activity compared to PAO1 harboring the empty vector pGM931. The absence of the molecular chaperone Hfq results in a lack of reporter activation by ErsA overexpression compared to wild-type. Significance by one-way ANOVA with *post-hoc* Tukey’s honestly significant difference is indicated: ^*^p<0.05; ^**^p<0.01; and ^***^p<0.001. **(C)** Comparison of the fluorescence polarization expressed in arbitrary units (AU) resulting from the translational fusion *anr::sfGFP* in PAO1 wild type (dark gray bars) and PAO1 *hfq^−^* (light gray bars) without ErsA overexpression. The lack of Hfq in the presence of ErsA levels that do not stimulate *anr::sfGFP* results in the reduction of the reporter activity. Statistical significance by t-test is indicated as: ^*^p<0.05. **(D)** *In vitro* interaction between ErsA RNA and *anr* mRNA by an electrophoretic mobility shift assay. 0.3pmol of ErsA RNA was incubated at 37°C for 20min with increasing amounts of *anr* RNA (0, 0.3, 0.6, 1.08, 2.1, and 4.2pmol; lanes 3–8) or, as a negative control, yeast tRNA (1 and 4pmol; lanes 1 and 2) and loaded into a native 6% polyacrylamide gel. Nucleic acids were transferred into GeneScreen plus nylon hybridization membranes. The sRNA/mRNA interactions were tested using biotinylated oligonucleotide probes pairing with the ErsA RNA. Free ErsA shows multiple electrophoretic forms. With the addition of the target RNA, free ErsA bands #1 and #2 disappear (black arrowheads #1 and #2), and other bands corresponding to the sRNA/mRNA complexes appear (red arrowheads #1 and #2).

**Figure 2:**
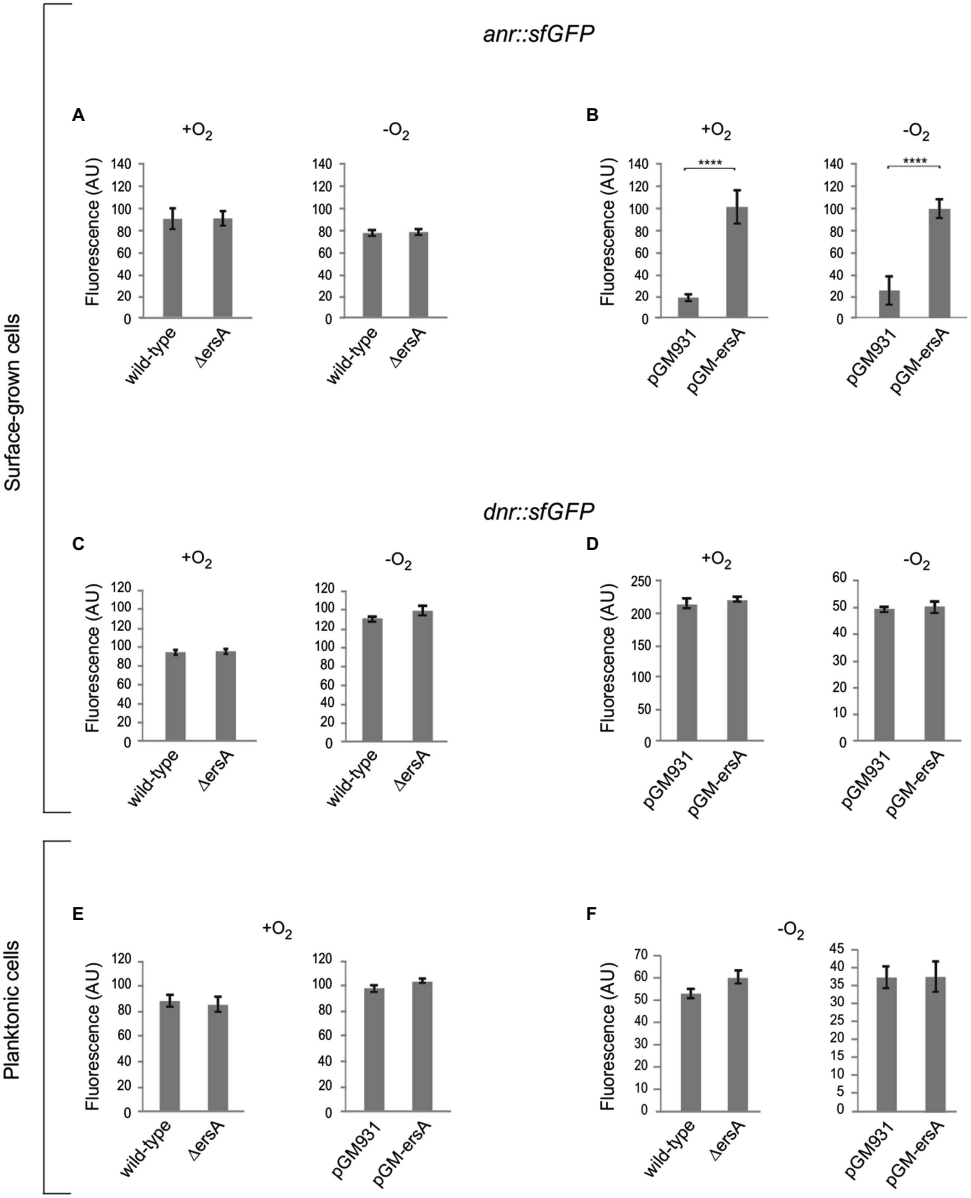
Analysis of the ErsA-mediated modulation of the *anr::sfGFP* and *dnr::sfGFP* translational fusions in surface-grown and planktonic cells, in both aerobic and anaerobic conditions. Fluorescence polarization in arbitrary units (AU) resulting from the translational fusions in PAO1 wild-type and Δ*ersA* strains, and in PAO1 strains with wild-type background harboring the pGM-ersA vector for ErsA overexpression, or the empty vector pGM931 are reported. **(A)** Comparison of the fluorescence resulting from the translational fusion *anr::sfGFP* in surface-grown PAO1 wild-type and Δ*ersA mutant cells*. **(B)** Comparison of the fluorescence resulting from the translational fusion *anr::sfGFP* in surface-grown PAO1 wild type harboring the pGM-*ersA* vector for ErsA overexpression, or the empty vector pGM931. **(C)** Comparison of the fluorescence resulting from the translational fusion *dnr::sfGFP* in surface-grown PAO1 wild-type and Δ*ersA mutant cells*. **(D)** Comparison of the fluorescence resulting from the translational fusion *dnr::sfGFP* in surface-grown PAO1 wild type harboring the pGM-*ersA* vector for ErsA overexpression, or the empty vector pGM931. **(E)** and **(F)** Comparison of the fluorescence resulting from the translational fusion *dnr::sfGFP* in planktonic PAO1 wild-type and Δ*ersA mutant cells*, and PAO1 wild type harboring the pGM-*ersA* vector for ErsA overexpression, or the empty vector pGM931, respectively. Statistical significance by *t*-test is indicated as: ^****^p<0.0001.

The results of the analysis described above suggested that *anr* and *dnr* mRNAs could be targeted by ErsA. To assess preliminarily the interaction between ErsA RNA and the two candidate target mRNAs, the web tool IntaRNA (Wright et al., 2014) was used. The modeling of the interaction between the full-length ErsA and *anr* mRNA comprehensive of 5' untranslated region (5'-UTR) predicted that the 34-nt long U-rich unstructured domain II of ErsA (Ferrara et al., 2015) could, from nt 31 to 54, extensively base-pair with the open reading frame region of *anr* mRNA, from nt +28 to +49 relative to the translational start site AUG (Figure 1A). To validate this sRNA/mRNA interaction *in vivo*, we used a robust two-plasmid reporter system suited for *P. aeruginosa* in our previous works (Ferrara et al., 2015, 2017). To this end, we generated a translational fusion between the first 22 codons of *anr* open reading frame linked to its 5'-UTR and the *superfolder* variant gene of the green fluorescent protein (*sfGFP*; Corcoran et al., 2012) under the control of the heterologous constitutive promoter *PLtetO-1*. We comparatively assayed the *anr::sfGFP* fusion in planktonic cells, grown in aerobic conditions, of wild-type and Δ*ersA* mutant strains and observed no significant differences in fluorescence levels. Then, the fluorescence expressed by the *anr::sfGFP* fusion was assayed in *P. aeruginosa* PAO1 strains, still grown in planktonic and aerobic conditions, in the absence and presence of ErsA overexpression from the arabinose inducible pGM-*ersA* vector (Ferrara et al., 2015). As shown in Figure 1B, ErsA overexpression by pGM-*ersA* conferred an approximately 2-fold increase in GFP activity compared to the strain harboring the control vector pGM931 (Qiu et al., 2008; Delvillani et al., 2014). Since spurious activating interactions of ErsA with the *sfGFP* open reading frame were ruled out (Ferrara et al., 2015), these results strongly suggested a positive direct modulation of *anr* mRNA by ErsA. Since a non-planktonic growth, e.g., adherent to a surface in form of either biofilm or colony can strongly influence *P. aeruginosa* physiology and gene expression, we assayed the *anr::sfGFP* fusion in *P. aeruginosa* cells grown as extended colonies on agar plates, either in aerobic or anaerobic conditions. As shown in Figure 2A, no significant differences in fluorescence levels of the *anr::sfGFP* fusion between wild-type and Δ*ersA* mutant strains were observed in both conditions. However, ErsA overexpression from the arabinose inducible pGM-*ersA* vector resulted in a significantly higher magnitude of induction of the *anr::sfGFP* fusion (Figure 2B) than the planktonic conditions (Figure 1B), namely, 5- (surface-grown cells) vs. 2-fold (planktonic cells). This enhanced effect of ErsA overexpression was independent of the presence of oxygen (Figure 2B). Overall, these results suggested that, above a certain abundance threshold, ErsA can enhance Anr mRNA translatability and the ErsA-mediated stimulation is more efficient when cells grow aggregated on a surface.

Furthermore, we aimed to evaluate whether the activity of the RNA chaperone Hfq could influence the ErsA-mediated activation of *anr* expression. This is because the regulatory activity of ErsA was shown to be dependent on Hfq for other targets (e.g., *algC*, *amrZ*, and *oprD*; Ferrara et al., 2015; Falcone et al., 2018; Sonnleitner et al., 2020) and it has been deduced that Hfq stimulates the expression of *anr* by an unknown mechanism since Anr was less expressed in an *hfq^−^* mutant of *P. aeruginosa* than in the corresponding wild type (Sonnleitner et al., 2011). The positive influence of Hfq on Anr expression was reconfirmed in our experimental system. As shown in Figure 1C, the fluorescence levels expressed by the translational fusion *anr::sfGFP* were significantly lower in the *hfq^−^* than in the wild-type background. These results suggested that Hfq plays *per se* a direct and positive post-transcriptional regulatory role on *anr*.

Besides, differently from the wild type, the overexpression of ErsA from pGM-*ersA* in an *hfq^−^* background (Sonnleitner et al., 2003) showed no effects on fluorescence levels generate by the *anr::sfGFP* fusion (Figure 1B). Despite Hfq contributes to ErsA stability (Ferrara et al., 2015), overexpression of ErsA from pGM-*ersA* in an *hfq^−^* strain reaches levels similar to those of the wild type (Ferrara et al., 2015). Therefore, the Anr activation failure by ErsA in the absence of effective Hfq was a genuine effect which strongly indicated that the ErsA-mediated activation of *anr* expression is an Hfq-dependent mechanism.

To assess *in vitro* the ErsA/*anr* mRNA interaction, the whole ErsA RNA and the *anr* mRNA region spanning −31 (5'-UTR) to +66 were synthesized *in vitro*, mixed, and analyzed on native polyacrylamide gels. We mixed fixed amounts of ErsA RNA with increasing concentrations of target *anr* mRNA. As shown in Figure 1D, ErsA showed multiple electrophoretic forms, with two prevalent fast-migrating bands, #1 and #2. After the addition of increasing amounts of the target *anr* mRNA, bands #1 and #2 progressively decreased in intensity, and bands corresponding to the sRNA/mRNA complexes appeared accordingly. No extra-bands formed when increasing amounts of control tRNAs preparation were mixed with ErsA RNA. This indicated that ErsA and *anr* RNAs can specifically interact.

We also modeled a possible interaction of ErsA with *dnr* mRNA. The IntaRNA tool predicted that an interval similar to the above nt of the unstructured region of ErsA could couple with the open read frame of *dnr*, from nt 16 to 38 downstream AUG start codon (Supplementary Figure S2). To test *in vivo* this prediction, we generated a *dnr::sfGFP* translational fusion cloning the whole 5'-UTR of the *dnr* gene and 96nt of its open reading frame, corresponding to the first 32 amino acids of Dnr, fused to *sfGFP*. As in the case of *anr*, we compared the fluorescence of the *dnr::sfGFP* reporter in surface-grown cells of the wild-type and Δ*ersA* strains under aerobic and anaerobic conditions, and no relevant differences were detected between the two strains (Figure 2C). However, differently from *anr::sfGFP*, ErsA overexpression had no effects on *sfGFP* expression (Figure 2D). We performed the same experiments with planktonic cells either in either aerobic or anaerobic conditions and again no effects of ErsA deletion or overexpression were detected, respectively (Figures 2E,F). Overall, these assays did not evidence any regulatory activity of ErsA on the translational fusion *dnr::sfGFP*.

### Effects of ErsA Regulation on *Anr* and *Dnr* Genes at the Transcription Level in Denitrification Conditions

The results presented above with the *anr::sfGFP* translational fusion revealed no decrease in the translation of Anr in the Δ*ersA* background under any oxygen conditions tested, either in planktonic or surface-grown cells. However, these tests may suffer from sensitivity when evaluating downregulations due to the stability of the reporter gene product vs. the detection of upregulation which is less affected by the half-life of the reporter. Therefore, to evaluate the ErsA-mediated regulation of Anr in physiological conditions of denitrification, we assessed whether the loss of ErsA and the ensuing expected decrease of translation rate could influence negatively the *anr* mRNA abundance in anaerobiosis. According to the well-characterized regulatory link between Anr and Dnr during anaerobic growth, i.e., the *dnr* gene is transcriptionally activated by Anr, the *dnr* mRNA levels were also expected to decrease in the Δ*ersA* background. On these bases, we evaluated the mRNA levels of both *anr* and *dnr* expressed under anaerobic conditions in surface-grown cells of the PAO1 wild-type and Δ*ersA* strains. Total RNAs were extracted from cell cultures grown for 3days in agar plates with BHI supplemented with 100mm KNO_3_ in a jar for anaerobiosis and analyzed by qRT-PCR. As shown in Table 2, the expression of *anr* showed a 1.26-fold decrease in the Δ*ersA* mutant relative to the wild-type strain. This was accompanied by a 1.53-fold decrease in *dnr* expression again in the Δ*ersA* mutant.

**Table 2:** Relative expression of *anr* and *dnr* in wild-type vs. Δ*ersA* PAO1 strains determined by qRT-PCR.

Strain	Relative expression^a^ (2^-ΔΔCT^)	
	*anr*	*dnr*
PAO1 wild type	1.00	1.00
PAO1 Δ*ersA*	0.79	0.65

a *The calculation of the relative expression of the anr and dnr genes in the ΔersA mutant vs. the WT strain was performed as described by the 2^−ΔΔCT^ method (Livak and Schmittgen, 2001; Vitale et al., 2008). Values represent the average of two independent experiments where qRT-PCR amplification of each sample was performed in (technical) triplicate reactions*

These results are consistent with positive ErsA-mediated regulation of Anr in denitrification conditions. According to the well-known effect that translation potential affects mRNA stability, it is conceivable that the *anr* mRNA is more prone to degradation because of lower translatability in the absence of ErsA. Lower levels of Anr would lead to a lower degree of activation of the transcription of *dnr* and thus to a decrease in the abundance of *dnr* mRNA.

### ErsA Plays a Critical Role in the Growth of a *CF*-Adapted *P. aeruginosa* Strain in Denitrification and Arginine Fermentation Conditions

**Figure 3:**
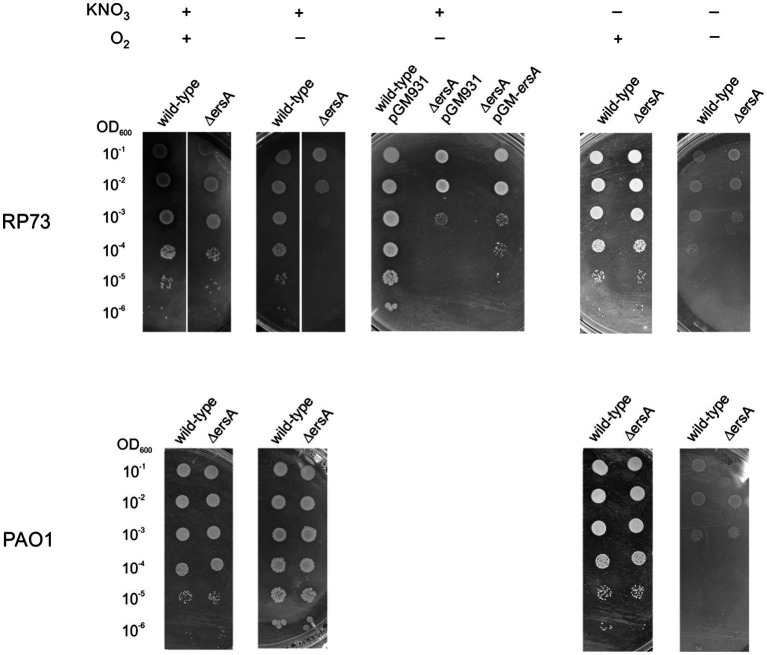
ErsA deletion impairs anaerobic growth in the RP73 clinical isolate. 2μl of cultures of the wild-type *P. aeruginosa* clinical isolate RP73 and PAO1 along with the corresponding ∆*ersA* mutants, serially diluted 10-fold, were spotted into BHI agar plates supplemented with 100mm KNO_3_ to allow anaerobic respiration or without KNO_3_ addition to assess arginine fermentation capacity. Plates were incubated under aerobic (+O_2_) or anaerobic (−O_2_) conditions. The presented results are representative of three independent experiments.

Although it was possible to detect the regulatory effects of ErsA on Anr and, subsequently, on Dnr during anaerobic respiration (Table 2), this ErsA role did not appear to be critical for *P. aeruginosa* PAO1 growth in these conditions. Since the experiments described above were not suited to quantitatively compare the growth efficiency between PAO1 wild-type and the Δ*ersA* mutants, we repeated the bacterial cell plating on BHI supplemented with KNO_3_ and incubated in anaerobiosis by spotting calibrated volumes of serial dilutions of quantified cell suspensions. We aimed also to assay in this way the other more virulent reference *P. aeruginosa* PA14 strain and the corresponding Δ*ersA* mutant (Ferrara et al., 2015). As anticipated, no substantial differences in growth rate and efficiency under denitrification conditions were detected between both PAO1 (Figure 3) and PA14 and their corresponding Δ*ersA* mutants. However, some important metabolic pathways occurring in low-oxygen conditions, such for instance denitrification, are perturbed by the competition for Hfq by the sRNA CrcZ, which can result in diminished anoxic growth and biofilm formation in *P. aeruginosa* (Pusic et al., 2016). It was indeed speculated that the Hfq sequestration-mediated function of CrcZ in limiting biofilm formation might be associated with the adaptive microevolution of *P. aeruginosa* for long-term persistence in the harsh environment of the *CF* airways (Pusic et al., 2016). Therefore, we wondered whether the regulatory effects of ErsA on Anr might be critical for the growth of a clinical strain of *P. aeruginosa* adapted for *CF*. To this end, we performed the calibrated bacterial cell plating on BHI supplemented with KNO_3_ and incubation in anaerobiosis comparing the *P. aeruginosa* multi-drug resistant clinical isolate RP73 (Bianconi et al., 2015) with the corresponding Δ*ersA* mutant strain (Ferrara et al., 2020). As shown in Figure 3, RP73 Δ*ersA* is strongly impaired in growing under denitrification conditions compared to wild type as evidenced by colonies extremely smaller and plating efficiency at least 10-fold lower. In the absence of KNO_3_ without oxygen, we also evaluated arginine fermentation for both PAO1 and RP73 and the corresponding Δ*ersA* mutants. As shown in Figure 3, differently from PAO1, the loss of ErsA in RP73 strongly impairs also arginine fermentation, the other energy process for which Anr is critical. Despite the *ersA* gene deletion in the *polA-engB* intergenic region shows no polar effects on flanking genes (Falcone et al., 2018) and the mutagenesis protocols that we used to generate it are suited to hinder secondary off-site mutations (Ferrara et al., 2015), we evaluated the effects on denitrification capacity of the RP73 Δ*ersA* strain following the reintroduction of the *ersA* gene expressed from pGM-*ersA*. As shown in Figure 3, pGM-*ersA* could complement the nitrate respiration in the RP73 Δ*ersA*, further supporting the notion that ErsA regulation is key for denitrification in the RP73 *CF*-adapted strain. Taken together, this suggested that *P. aeruginosa* adaptation to *CF* lung might result in a higher dependence on ErsA for the regulation of anaerobic energy metabolism.

## Discussion

**Figure 4:**
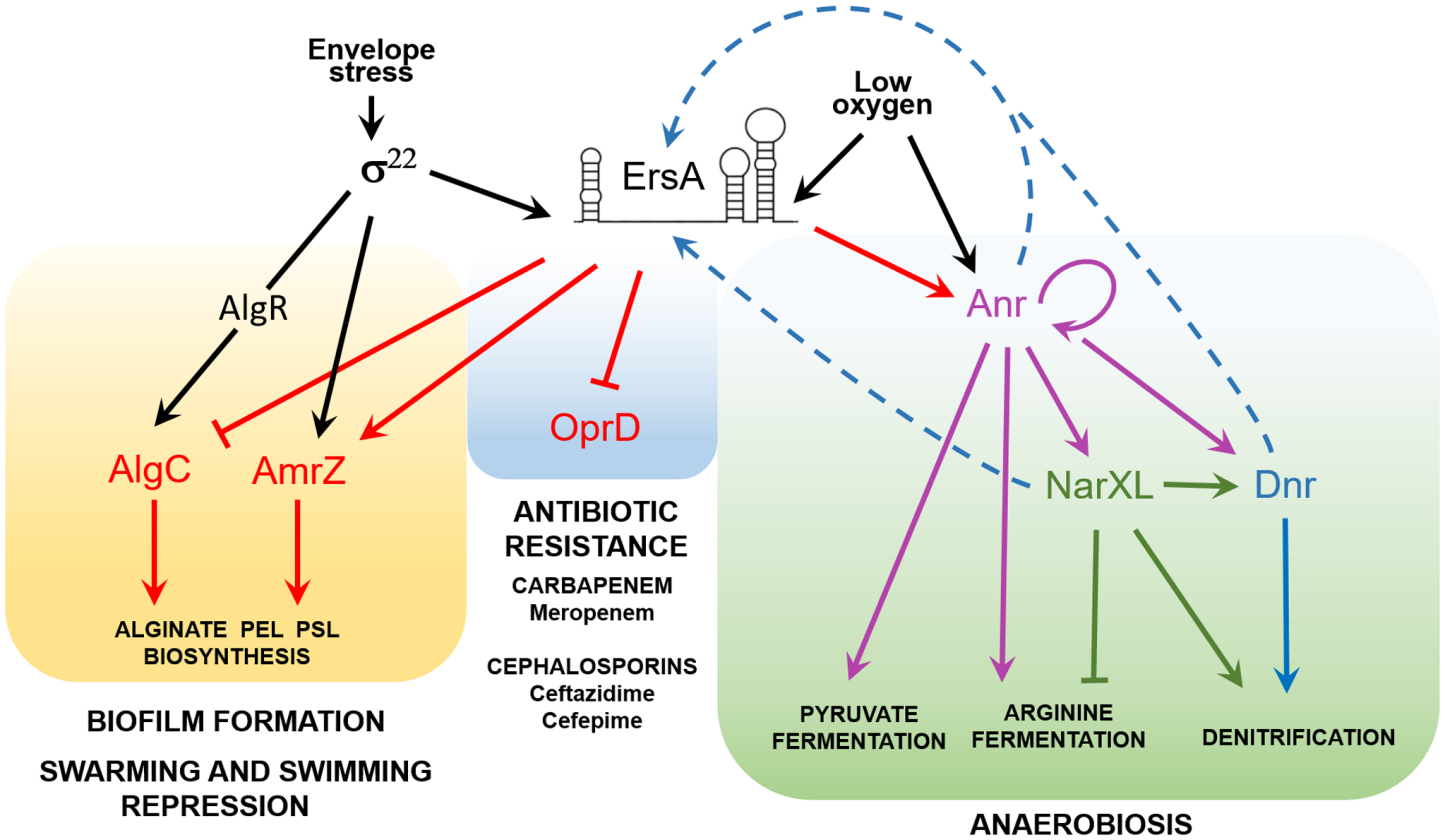
The ErsA regulatory network in *P. aeruginosa*. Acting as a post-transcriptional modulator, ErsA influences several virulence traits of *P. aeruginosa*, related to biofilm formation, repression of swarming and twitching motility, antibiotic resistance, and metabolic adaptation to low-oxygen environments typical of *CF* lung infection. ErsA modulates alginate and exopolysaccharides (Pel and Psl) production by activating the AmrZ regulon and through the fine-tuning of AlgC levels. The regulatory circuit is mediated by the envelope stress-responsive sigma factor σ^22^, which in *P. aeruginosa* triggers ErsA levels and is responsible for the transcription of *amrZ* mRNA and the activation of *algC* through the regulator AlgR. ErsA negatively regulates the expression of the major porin *oprD*, responsible for the uptake of carbapenem antibiotics, thus leading to increased resistance to meropenem. By unknown mechanisms, ErsA contributes also to ceftazidime and cefepime resistance, two antibiotics that belong to the class of cephalosporins. ErsA modulates the expression of genes for anaerobic growth and survival. Low-oxygen levels trigger the transcription of ErsA, which positively modulates the expression of the transcription factor Anr, the master regulator of anaerobiosis. Activation of Anr by dimerization is also promoted by low-oxygen tension. Other genes involved in anaerobic adaptation are also regulated by ErsA by an unknown mechanism. The signal that activates ErsA expression under an anaerobic or low-oxygen environment is still unknown. We speculate that ErsA expression can be controlled by transcription factors like Anr, Dnr, or NarXL (blue dotted arrows). A putative NarL box (TACCGCT) is present from 179 to 173nt upstream of the *ersA* transcription start site.

The sRNA ErsA is inducible by different cues relevant for airway infection, such as, for instance, envelope stress and shift from aerobic to anaerobic conditions (Figure 4). This environmental response is translated by ErsA in the modulation of biofilm dynamics through the activation of the AmrZ regulon (Falcone et al., 2018), the repression of a crossroad, the enzyme AlgC, of important pathways for the biosynthesis of sugar precursor leading to exopolysaccharides production (Ferrara et al., 2015), and of carbapenem resistance, *via* the negative regulation of porin OprD (Zhang et al., 2017). For its role in biofilm regulation, ErsA has been implicated as an important player in the regulatory network of *P. aeruginosa* pathogenicity in airway infection (Ferrara et al., 2020). With this work, we add a new piece to the ErsA role of transducing environmental signals into physiological responses showing that ErsA positively regulates Anr levels. Since Anr is the master regulator of the anaerobic response of *P. aeruginosa*, with a regulon of approximately 170 predicted transcription units (Trunk et al., 2010), ErsA rises to the role of a key co-mediator of the *P. aeruginosa* anaerobic metabolism.

Furthermore, this work highlights how ErsA can constitute a flexible regulatory node linked to the adaptive plasticity of *P. aeruginosa*. This is evidenced by the fact that the lack of ErsA dramatically impacts the possibility to grow by anaerobic nitrate respiration and arginine fermentation of a strain adapted to the *CF* lung environment, RP73, and not of reference strains, such as PAO1 and PA14. This phenomenon is coupled with the results of our previous work where the lack of ErsA in RP73 induced sensitivity to the antibiotics ceftazidime, cefepime, meropenem, and ciprofloxacin, suggesting that ErsA could contribute to *P. aeruginosa* adaptation to long-term antibiotic treatment undergone by *CF* patients. Dynamicity of the regulatory networks of bacterial functions for long-term persistence in the *CF* lung environment is frequently observed during the adaptive radiation of *P. aeruginosa* in such context where bacteria endure various attacks, encompassing oxidative stresses, immune responses, and prolonged antibiotic treatments. Our results suggest that the genetic adaptation to *CF* lung occurred in the strain RP73 might have led to a higher dependence on ErsA for the transduction of the multiple signals to the regulatory network of key functions for survivance in such a complex environment. In addition, other factors linked to ErsA might be critical for denitrification regulation in the *CF*-adapted RP73 and not in the reference strain PAO1.

From a mechanistic point of view, we can speculate that ErsA expression in response to environmental factors, such as anaerobiosis and possibly other cues in *CF* lungs, can be modulated to compensate for physiological adaptations involving a decrease in the amounts of effective Hfq, which is known to have a large regulon (Sonnleitner et al., 2006) and a pivotal role in *P. aeruginosa* physiology (anaerobic metabolism included) and virulence (Sonnleitner et al., 2003; Pusic et al., 2016). A key regulator impacting the abundance of effective Hfq is the sRNA CrcZ that acts as a decoy to abrogate Hfq-mediated translational repression of catabolic genes (Sonnleitner and Blasi, 2014) and indeed mediating the carbon catabolite repression (CCR) mechanism in *P. aeruginosa* but also implementing Hfq sequestration for the cross-regulation of the panoply of Hfq-dependent physiological processes (Pusic et al., 2016). For example, high levels of CrcZ and therefore low abundance of active Hfq were evoked to explain the limitation of anoxic biofilm formation and this scenario could occur in the adaptation of *P. aeruginosa* to the *CF* lung (Pusic et al., 2016). In this context, the regulatory node of ErsA could co-adapt and become critical to compensate for the limiting amount of effective Hfq.

The mediating role of Hfq in anaerobic metabolism appears to be large and important. The absence of Hfq results in an increased abundance of transcripts encoded by the *nar*, *nap*, and *nor* operons, encoding enzymes required for denitrification. Besides, several *nuo* transcripts, encoding subunits of the NADH dehydrogenase, were downregulated in the absence of Hfq (Pusic et al., 2016). Incidentally, the NADH dehydrogenase is required for anaerobic growth in the presence of nitrate, contributes to the intracellular redox balance, i.e., the NADH/NAD^+^ ratio, and is linked to the energizing processes of the membrane and ATP synthesis. Furthermore, it was reported that Hfq stimulates the expression of *anr* by an unknown mechanism (Sonnleitner et al., 2011).

Our results strongly suggest that this regulation is directly given by Hfq at the post-transcriptional level. Furthermore, beyond a certain threshold of abundance, we show that ErsA participates in the positive regulation of Anr through an Hfq-dependent mechanism. Also for *anr* mRNA, the role of Hfq chaperone could be that of favoring the ErsA/*anr* mRNA interaction pathway which may result in enhanced translatability of the *anr* mRNA due, for example, to the increased accessibility of the translation start site. This positive ErsA-mediated effect would be a component of the *anr* mRNA stabilization and consistent with the decrease in *anr* mRNA abundance in the lack of ErsA. Besides, we can postulate an additional interplay between ErsA and Hfq in the post-transcriptional regulation of Anr based on reciprocal recruitment on *anr* mRNA which might stabilize the transcript and participates further in the positive effects on Anr translation.

Therefore, this work suggests the correlation between the property of ErsA of being transcriptionally activated under reduced oxygen conditions (Ferrara et al., 2015), and its role in participating in the transduction of the low-oxygen cue toward the Anr regulon, acting as a positive post-transcriptional regulator of the *anr* mRNA. However, other environmental signals modulating ErsA could be important in the fine-tuning of the Anr regulon.

From the point of view of the *P. aeruginosa* anaerobic lifestyle during airways infection, it was shown that a Δ*anr* mutant is attenuated in a mouse pneumonia model of acute infection (Jackson et al., 2013). Besides, the *anr* gene deletion leads to defective biofilm formation, while increased Anr activity results in enhanced biofilm formation (Jackson et al., 2013). Despite the differences in the experimental setting of infections, the strong attenuation of acute infection that we reported for the PAO1 Δ*ersA* mutant (Ferrara et al., 2020) is consistent with the *P. aeruginosa* behavior in the absence of Anr described previously. The lack of ErsA-mediated regulation of several factors, including Anr, could prevent the formation of biofilm-like aggregates that assemble on airway surfaces (Tran et al., 2014) and promote mucosal colonization leading to bypassing the epithelial barrier and thus invasion and systemic dissemination (Sadikot et al., 2005).

In this work, we also propose that the role of ErsA in regulating the anaerobic physiology of *P. aeruginosa* is broader than the upstream co-modulation of Anr regulon, whose most important regulatory member is undoubtedly Dnr. Indeed, the transcriptional profile of the *ersA* mutant also shows a dysregulation of genes not belonging to Anr regulon but still involved in anaerobic metabolism (Falcone et al., 2018). It is therefore plausible that ErsA exerts other regulatory functions, acting as a direct or indirect regulator of the genes listed in Table 1. Other unlisted genes may also be direct targets that have not been detected due to a “below-threshold” concentration of ErsA in the wild-type strain or to unimpaired mRNA stability in the *ersA* mutant. The *algC* gene is a representative example of the second case, whose mRNA levels are comparable in the *ersA* mutant and the wild type (Ferrara et al., 2015).

In summary, the list of known ErsA-regulated genes is becoming more and more populated (Figure 4) and with this work, it has extended to anaerobic metabolism. It is evident that important ErsA-modulated *P. aeruginosa* phenotypes are related to both acute and chronic airway infection and, associated with the latter, to adaptive microevolution in the *CF* environment.

## Data Availability Statement

The raw data supporting the conclusions of this article will be made available by the authors, without undue reservation.

## Author Contributions

GB and SF conceived, designed the study, and wrote the paper. SF, RC, SS, and GB conceived the experiments and analyzed the data. SF, RC, and SS designed and performed the experiments. All authors contributed to the article and approved the submitted version.

## Conflict of Interest

The authors declare that the research was conducted in the absence of any commercial or financial relationships that could be construed as a potential conflict of interest.

## Publisher’s Note

All claims expressed in this article are solely those of the authors and do not necessarily represent those of their affiliated organizations, or those of the publisher, the editors and the reviewers. Any product that may be evaluated in this article, or claim that may be made by its manufacturer, is not guaranteed or endorsed by the publisher.
