# Supplementary material for: The Small RNA ErsA Impacts the Anaerobic Metabolism of Pseudomonas aeruginosa Through Post-Transcriptional Modulation of the Master Regulator Anr
Source: Front Microbiol. 2021 Aug 20;12:691608. doi: 10.3389/fmicb.2021.691608 (PMC8575079; doi:10.3389/fmicb.2021.691608)
Supplement: Supplementary file 1 [file Table_1.pdf]

Table S1. Strains and plasmids

Strains or plasmids	Genotype or description	Construction	Reference
Pseudomonas aeruginosa			
PAO1	Wild-type		(Stover et al., 2000)
PAO1 *hfq-*	*hfq::aadA*		(Sonnleitner et al., 2003)
PAO1 Δ*ersA*	markerless Δ*ersA*		(Ferrara et al., 2015)
P. aeruginosa RP73	Wild-type		(Bianconi et al., 2015)
P. aeruginosa RP73 Δ*ersA*	markerless Δ*ersA*		(Ferrara et al., 2020)
Escherichia coli			
TOP10	mcrA Δ(mrr-hsdRMS-mcrBC) φ80lacZDM15 ΔlacX74 deoR recA1 araD139 Δ(ara-leu)7697 galU galK rpsL endA1 nupG		Invitrogen
Plasmids			
pGM931	pHERD20T derivative, *araC*/P_{BAD} - t_{Ω}, Ap^r		(Qiu et al., 2008; Delvillani et al., 2014; Ferrara et al., 2015)
pGM-*ersA*	pGM931 derivative, *ersA* under P_{BAD}, Ap^r		(Ferrara et al., 2015)
pXG10-SF	sfGFP reporter plasmid; *lacZ*::*gfp* under $P_{LtetO-1}$, Cm^r		(Corcoran et al., 2012)
pXG10-*anr::sfGFP*	pXG10-SF derivative; $P_{LtetO-1}$ -->*anr::gfp*, Cm^r	contains PCR product using oligos 2 and 3	this work
pXG10-*dnr::sfGFP*	pXG10-SF derivative; $P_{LtetO-1}$ -->*dnr::gfp*, Cm^r	contains PCR product using oligos 4 and 5	this work
pBBR1-MCS5	*lacZ* α, Gm^r		(Kovach et al., 1995)
pBBR1- *sfGFP*	pBBR1-MCS5 derivative; $P_{LtetO-1}$ -->*gfp*, Gm^r		(Ferrara et al., 2015)
pBBR1-*anr::sfGFP*	pBBR1-MCS5 derivative; $P_{LtetO-1}$ -->*anr::gfp*, Gm^r	contains PCR product using oligos 6 and 7	this work
pBBR1-*dnr::sfGFP*	pBBR1-MCS5 derivative; $P_{LtetO-1}$ -->*dnr::gfp*, Gm^r	contains PCR product using oligos 6 and 7	this work

References

Bianconi, I., Jeukens, J., Freschi, L., Alcala-Franco, B., Facchini, M., Boyle, B., et al. (2015). Comparative genomics and biological characterization of sequential Pseudomonas aeruginosa isolates from persistent airways infection. *BMC Genomics* 16**,** 1105. doi: 10.1186/s12864-015-2276-8.

Corcoran, C.P., Podkaminski, D., Papenfort, K., Urban, J.H., Hinton, J.C., and Vogel, J. (2012). Superfolder GFP reporters validate diverse new mRNA targets of the classic porin regulator, MicF RNA. *Mol Microbiol* 84(3)**,** 428-445. doi: 10.1111/j.1365-2958.2012.08031.x.

Delvillani, F., Sciandrone, B., Peano, C., Petiti, L., Berens, C., Georgi, C., et al. (2014). Tet-trap, a genetic approach to the identification of bacterial RNA thermometers: Application to Pseudomonas aeruginosa. *RNA* 20(12)**,** 1963-1976. doi: 10.1261/rna.044354.114.

Ferrara, S., Carloni, S., Fulco, R., Falcone, M., Macchi, R., and Bertoni, G. (2015). Post-transcriptional regulation of the virulence-associated enzyme AlgC by the σ22-dependent small RNA ErsA of Pseudomonas aeruginosa. *Environmental Microbiology* 17(1)**,** 199-214. doi: 10.1111/1462-2920.12590.

Ferrara, S., Rossi, A., Ranucci, S., De Fino, I., Bragonzi, A., Cigana, C., et al. (2020). The Small RNA ErsA Plays a Role in the Regulatory Network of Pseudomonas aeruginosa Pathogenicity in Airway Infections. *mSphere* 5(5). doi: 10.1128/mSphere.00909-20.

Kovach, M.E., Elzer, P.H., Hill, D.S., Robertson, G.T., Farris, M.A., Roop, R.M., 2nd, et al. (1995). Four new derivatives of the broad-host-range cloning vector pBBR1MCS, carrying different antibiotic-resistance cassettes. *Gene* 166(1)**,** 175-176.

Qiu, D., Damron, F.H., Mima, T., Schweizer, H.P., and Yu, H.D. (2008). PBAD-based shuttle vectors for functional analysis of toxic and highly regulated genes in Pseudomonas and Burkholderia spp. and other bacteria. *Appl Environ Microbiol* 74(23)**,** 7422-7426. doi: AEM.01369-08 [pii]

10.1128/AEM.01369-08.

Sonnleitner, E., Hagens, S., Rosenau, F., Wilhelm, S., Habel, A., Jager, K.E., et al. (2003). Reduced virulence of a hfq mutant of Pseudomonas aeruginosa O1. *Microb Pathog* 35(5)**,** 217-228. doi: 10.1016/s0882-4010(03)00149-9.

Stover, C.K., Pham, X.Q., Erwin, A.L., Mizoguchi, S.D., Warrener, P., Hickey, M.J., et al. (2000). Complete genome sequence of Pseudomonas aeruginosa PAO1, an opportunistic pathogen. *Nature* 406(6799)**,** 959-964. doi: 10.1038/35023079.
