# Supplementary material for: The Small RNA ErsA Impacts the Anaerobic Metabolism of Pseudomonas aeruginosa Through Post-Transcriptional Modulation of the Master Regulator Anr
Source: Front Microbiol. 2021 Aug 20;12:691608. doi: 10.3389/fmicb.2021.691608 (PMC8575079; doi:10.3389/fmicb.2021.691608)
Supplement: Supplementary file 2 [file Table_2.pdf]

Table S2. Oligonucleotides

Oligo #	Oligo Name	Sequence (5' → 3')[a]	Utilization and Reference
1	5'BIOTIN-SPA122	[BIO]CCCGAGCTTCGTATGGGG	Biotinylated Northern-blot probe for ErsA (modified from (Ferrara et al., 2012)
2	-31ATG_anr_NsiI_for	GTTTTatgcatTGTTGTGCCTTTAACCTAGCAAG	Amplification of the 5' UTR and beginning of *anr* with *Nsi*I/*Nhe*I ends for cloning in frame with *gfp* in pXG10-SF vector
3	+66ATG_anr_NheI_rev	GTTTTgctagcCGGGGCCAGACTGCAATC	
4	-116ATG_dnr_NsiI_for	GTTTTatgcatTCCGCTCGCCGGACCGAC	Amplification of the 5' UTR and beginning of *dnr* with *Nsi*I/*Nhe*I ends for cloning in frame with *gfp* in pXG10-SF vector
5	+96ATG_dnr_NheI_rev	GTTTTgctagcGCTGCTGGCGAGCAGTTC	
6	Ptet-O1_ClaI_for	GTTTTatcgatTCCCTATCAGTGATAGAG	Amplification from the $P_{LtetO\text{-}1}$ promoter to the stop codon of *gfp* of pXG10-*anr*::*gfp*/ pXG10-*dnr*::*gfp* with *Cla*I/*Xba*I ends for cloning in pBBR1-MCS5 (Ferrara et al., 2015)
7	sfGFP_TAA_XbaI_rev	TGATGCCtctagaTTATTTGTAGAGCTC	
8	sfGFP_+96_rev	TTGTGCCCATTAACATCACCATC	Reverse primer on *gfp* for verification of constructs (Ferrara et al., 2015)
9	T7_-31ATG_anr_for	**CTAATACGACTCACTATAGGG**TGTTGTGCCTTTAACCTA	Amplification of the 5' UTR and beginning of *anr* in fusion with RNA polymerase T7 promoter for *in vitro* transcription
10	+66ATG_anr_rev	CGGGGCCAGACTGCAATC	
11	T7_5'SPA0122_for	**CTAATACGACTCACTATAGGG**CGAATGGCTTCTTGAGCC	Amplification of *ersA* in fusion with RNA polymerase T7 promoter for *in vitro* transcription (Ferrara et al., 2015)
12	3'SPA0122_rev	AAAAAAAACCCCGAGCTTCGTATGGGGAG	
13	16S_for	TGTCGTCAGCTCGTGTCGTGA	Amplification of 16S for real time-PCR analysis (Vitale et al., 2008)
14	16S_rev	ATCCCCACCTTCCTCCGG T	
15	qAnr_for	GCCCAGGCCCTGGAAAC	Amplification of *anr* for real time-PCR analysis (Tribelli et al., 2019)
16	qAnr_rev	ATCATCTGCTGGTCATCGCG	
17	qDnr_For	AGCCAGCTGTTCCGTTTCTC	Amplification of *dnr* for real time-PCR analysis (Jackson et al., 2013)
18	qDnr_Rev	GTGGCGTTCTTCAGGGAAAG	

[a] [BIO]: Biotin. Lowercase: sites for restriction enzymes. Bold: T7 promoter sequence.

References

Ferrara, S., Brugnoli, M., de Bonis, A., Righetti, F., Delvillani, F., Dehò, G., et al. (2012). Comparative profiling of pseudomonas aeruginosa strains reveals differential expression of novel unique and conserved small RNAs. *PLoS ONE* 7(5). doi: 10.1371/journal.pone.0036553.

Ferrara, S., Carloni, S., Fulco, R., Falcone, M., Macchi, R., and Bertoni, G. (2015). Post-transcriptional regulation of the virulence-associated enzyme AlgC by the σ22-dependent small RNA ErsA of Pseudomonas aeruginosa. *Environmental Microbiology* 17(1)**,** 199-214. doi: 10.1111/1462-2920.12590.

Jackson, A.A., Gross, M.J., Daniels, E.F., Hampton, T.H., Hammond, J.H., Vallet-Gely, I., et al. (2013). Anr and its activation by PlcH activity in Pseudomonas aeruginosa host colonization and virulence. *J Bacteriol* 195(13)**,** 3093-3104. doi: 10.1128/JB.02169-12.

Tribelli, P.M., Lujan, A.M., Pardo, A., Ibarra, J.G., Fernandez Do Porto, D., Smania, A., et al. (2019). Core regulon of the global anaerobic regulator Anr targets central metabolism functions in Pseudomonas species. *Sci Rep* 9(1), 9065. doi: 10.1038/s41598-019-45541-0.

Vitale, E., Milani, A., Renzi, F., Galli, E., Rescalli, E., De Lorenzo, V., et al. (2008). Transcriptional wiring of the TOL plasmid regulatory network to its host involves the submission of the σ54-promoter Pu to the response regulator PprA. *Molecular microbiology* 69(3), 698-713.
