# Supplementary material for: The Small RNA ErsA Impacts the Anaerobic Metabolism of Pseudomonas aeruginosa Through Post-Transcriptional Modulation of the Master Regulator Anr
Source: Front Microbiol. 2021 Aug 20;12:691608. doi: 10.3389/fmicb.2021.691608 (PMC8575079; doi:10.3389/fmicb.2021.691608)
Supplement: Supplementary file 3 [file Image_1.pdf]

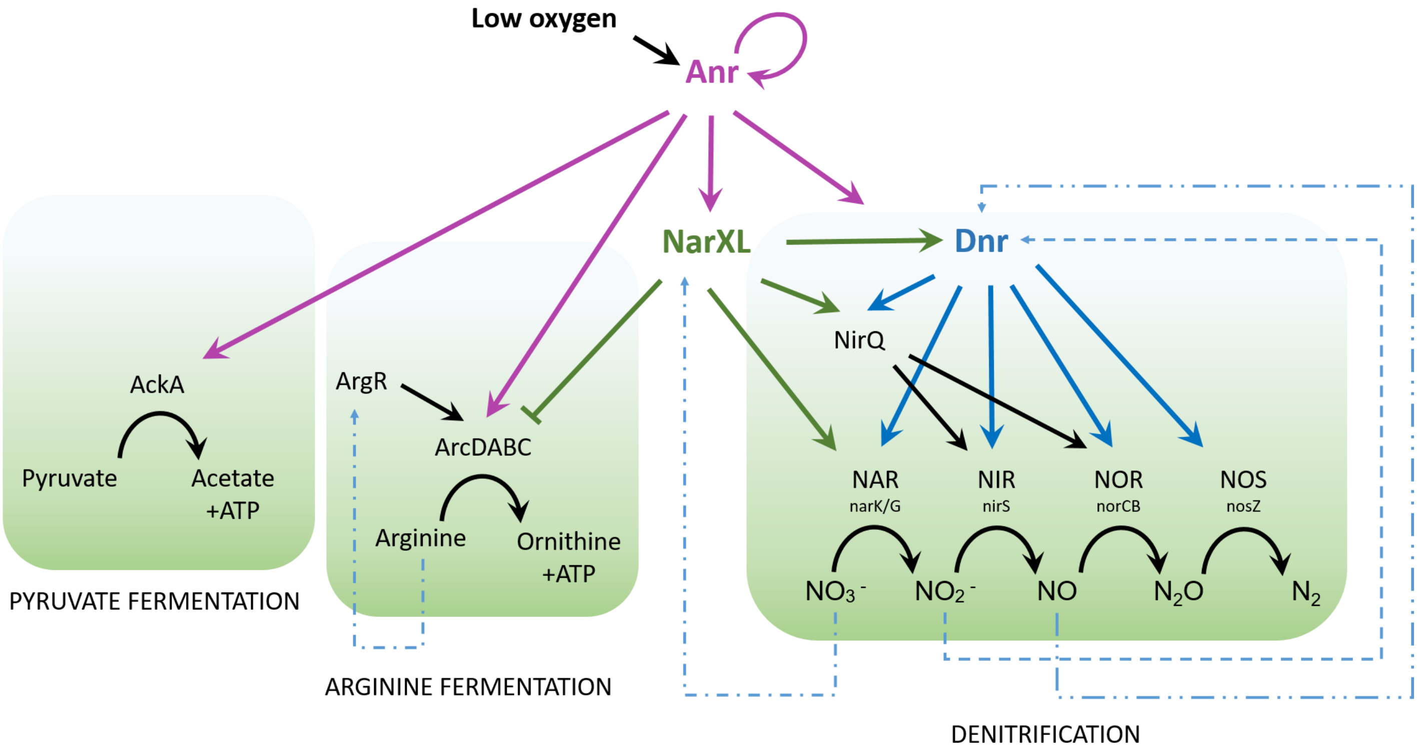


**Figure S1.** Model of the regulatory network controlling the anaerobiosis-associated genes in *P. aeruginosa*. Under anaerobic or low oxygen conditions, the transcription factor Anr undergoes activation by conformational changes that allow its dimerization. Once activated, Anr positively controls its expression, and the expression of NarXL, a two-component system responding to the presence of nitrate and required, together with Anr, for expression of Dnr. The transcription factor Dnr is responsive to the presence of nitrate and acts with NarXL for the activation of NirQ. Dnr activates the expression of all denitrification genes (NAR, NIR, NOR, and NOS) in response to nitric oxide.

Activated Anr also regulates the *arcDABC* operon for arginine fermentation. In presence of arginine, the *arcDABC* operon is also stimulated through the ArgR regulator. When arginine and nitrate are both present, NarXL competes with ArgR, and represses the *arcDABC* operon, promoting the denitrification pathway. Finally, Anr positively regulates the transcription of the *ackA-pta* operon involved in pyruvate fermentation. In the absence of nitrate, nitrite, or arginine, the acetate kinase AckA utilized pyruvate to produce acetate and ATP. This reaction allows survival but not the growth of *P. aeruginosa* cells during severe anaerobic energy starvation conditions.
