# Supplementary material for: The Small RNA ErsA Impacts the Anaerobic Metabolism of Pseudomonas aeruginosa Through Post-Transcriptional Modulation of the Master Regulator Anr
Source: Front Microbiol. 2021 Aug 20;12:691608. doi: 10.3389/fmicb.2021.691608 (PMC8575079; doi:10.3389/fmicb.2021.691608)
Supplement: Supplementary file 4 [file Image_2.pdf]

```
16  GUCCACCAGCAGU---UGCUGCAAAG  38  dnr mRNA
    :||  |||:|| |   ::|||||| |
56  UAGACGGUUGUGAUUUGUGACGUUUC  31  ErsA
```

Figure S2. Model of base-pairing interaction between ErsA and *dnr* mRNA by IntaRNA software. The region from nt +31 to +56 of ErsA was predicted to bind within the coding sequence of *dnr* mRNA, from nt 16 to nt 38 downstream the translation start site (ATG). Sequence coordinates refer to the +1 translational start site for *dnr* mRNA.
